# Validation of drop plate technique for bacterial enumeration by parametric and nonparametric tests

**Published:** 2013

**Authors:** Hossein Naghili, Hossein Tajik, Karim Mardani, Seyed Mehdi Razavi Rouhani, Ali Ehsani, Payman Zare

**Affiliations:** 1 *Department of Food Hygiene and Quality Control, Faculty of Veterinary Medicine, Urmia University, Urmia, Iran; *; 2 *Department of Pathobiology, Faculty of Veterinary Medicine, University of Tabriz, Tabriz, Iran.*

**Keywords:** Drop plate, *Lactobacillus casei*, Parametric and nonparametric tests, S*almonella *Typhimurium, Spread plate

## Abstract

Drop plate technique has a priority and preference compared with the spread plate procedure, because of less time, quantity of media, effort requirement, little incubator space, and less labor intensive. The objective of this research was to compare the accuracy and fidelity of drop plate method vs. spread plate method by parametric and nonparametric statistical tests. For bacterial enumeration by drop and spread plate methods, successive dilutions of second subculture of *Lactobacillus casei* and *Salmonella* Typhimurium were transferred to selective agar. The correlation of agreement between both methods was evaluated by using statistical proofs. Results showed that mean value (parametric unpaired *t*-test) comparison at 95 percent confidence level did not reject null hypothesis, which it meant that the equality of the mean data could not be ruled out. Nonparametric method was used because of approximately Gaussian pattern of data distribution. For this purpose, Mann-Whitney test (equivalent nonparametric *t*-test) was used. It meant that the equality of medians obtained from two methods were similar. Spearman’s rho correlation coefficient (r) via both methods due to data distribution patterns for enumeration of *S.* Typhimurium and *L. casei* were 0.62 and 0.87, respectively; which represented moderately strong and strong relationship between two methods, respectively. Besides, there was a significant and strong positive correlation (*p* < 0.001) between spread and drop plate procedures. Because of aforementioned reasons, the spread plate method can be replaced by drop plate method.

## Introduction

Some methods are found application in bacterial enumeration of milk, foods and cosmetics like microscopic scanning of stained organisms, measurement of various enzyme concentrations, spiral plate, quantitative real-time PCR, etc. Among conventional methods (pour plate, spread plate method) drop plate technique (DP) has been utilized routinely because of their less expenditure and equipment. 

The DP is being exploited in numerous laboratories across the world. In spite of its widespread applicability, the DP has not been standardized.^1^^,^^2^ Numerous papers have compared the accuracy of enumeration methods. Whereby there is not a standardized procedure for the size of the drops (10-30 µL per drop), the number of replications, or the number of sectors (dilutions) used per plate.^1^

The DP method is a mixture of microbiological components and design components. The microbiological factors should have been tuned up for different bacteria. They include the bacterial species, strains and growth conditions e.g., media, agar, temperature, time.^3^ In fact the DP has a miniaturized version of spread method (SP) method. In this investigation we tried to take advantage of parametric and nonparametric tests to show that SP could be replaced with DP as an alternative method.

## Materials and Methods 

Bacterial strains and culture condition* Lactobacillus casei* (ATCC 39392) and *Salmonella *Typhimurium LT_2 _(ATCC 700792) were obtained from the culture collection of the Department of Pathobiology, Faculty of Veterinary Medicine, University of Tabriz, Tabriz, Iran.

**Fig. 1:**
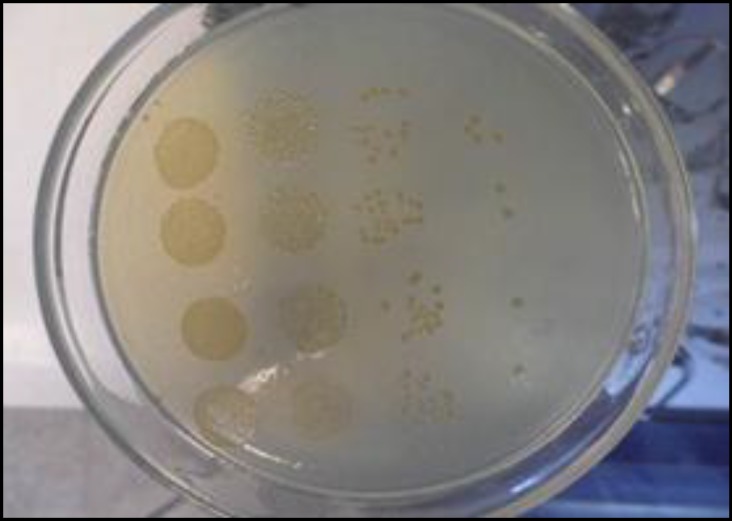
The DP method for enumeration of* S. *Typhimurium. The dose of spots from left to right corresponded to 4, 5, 6, 7 log_10_* S. *Typhimurium.

The bacteria were maintained in bead containing cryo-tubes at -70 ˚C. Working inoculums of bacteria were prepared by transferring a bead containing bacteria to the brain heart infusion (BHI) agar (Himedia, Mumbai, India) and/or De Mann, Rogosa and Sharp (MRS) agar (Merck, Darmstadt, Germany) slant tubes for *S. *Typhimurium and *L. casei*, respectively and incubated at 37 ± 1 ˚C for 24 hr. Prior to the empirical test, the bacteria were reactivated by two subcultures. For first subculture, three or four well-isolated colonies were touched with a sterile wire loop and suspended into 10 mL of Luria-Bertani (LB) broth (Biomark, Pune, India) and MRS broth (Merck, Darmstadt, Germany), for *S*. Typhimurium and *L. casei *respectively and incubated at 37 ± 1 ˚C for 24 hr with continuous agitation at 150 rpm. Subsequently, a second subculture was prepared and incubated for 20 hr at 37 ± 1 ˚C as well. Bacterial suspensions adjusted to approximately desired log_10_ CFU mL^−1^ by ultraviolet-visible spectrophotometry at 600 nm then by using standard serial 10-fold dilution in buffered peptone water (Merck, Germany) and eventually transferred 10 and 100 µL for drop and spread plating on bismuth sulfite agar (BSA) (Merck, Darmstadt, Germany) and MRS agar, for *S. *Typhimurium and *L. casei, *respectively. The drops were absorbed to agar in less than half an hour. After the drops on the agar absorbed, the plates were incubated at inverted positions.^2^ Enumeration of* S. *Typhimurium viable cells were done after 17-20 hr at 30 ± 1 ˚C in aerobic incubation and for *L. casei *after 48 hr at 37 ± 1 ˚C in a 5% CO_2_ atmosphere.^4^ At least 3 to 30 colonies grew from 10 µL of drop and 30 to 300 CFU per 100 µL of SP as a confidence technique were chosen to count by using colony counter Funke-Gerber GMBH, Nr. 2774, Berlin/Munchen (Fig. 1). We averaged the total count of CFU over all at least 3 drops at the countable dilution. Finally, the total count was scaled up and the viable cell counts were expressed as CFU mL^-1^.^5^

**Statistical analysis. **For analyses and graphical presentations, Graphpad prism^®^ Software (version 5.04, San Diego, CA, USA) and MINITAB (version 16/2/0, Minitab Inc, State College, Pa, USA) were used. Each dilution was plated in duplicate with four drops per plate. A significant difference was considered as *p* < 0.05.

## Results

**Fig. 2:**
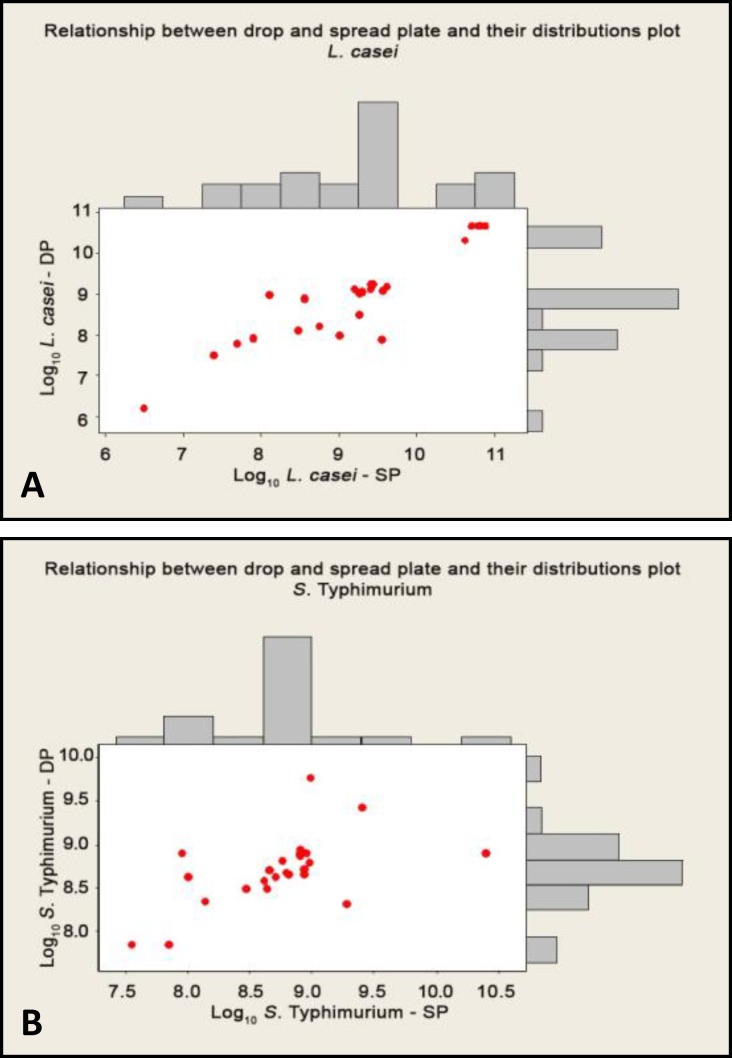
A marginal plot demonstrated a scatterplot with histograms of the x and y variables in the margins. This two-in-one graph compared variables and their distributions at the same time. **A)** *L. casei ****B*)*** S. *Typhimurium.

The distribution histogram of data represent approximate a Gaussian distribution. Relationship between DP and SP methods and their distributions among aforementioned bacteria were shown in marginal plots (Fig. 2).

**Parametric analysis. **Although the parametric test is based on the assumption that the data are normally distributed, this assumption is not important when the sample sizes are at least 15 or 20. It showed that the difference of *L. casei* and *S. *Typhimurium countable means between DP and SP methods by unpaired *t*-test (parametric) were 0.4256 and 0.8867, respectively. The *p*-values (0.4256 and 0.8867) for counting indicated that there was sufficient evidence that all the means were equal when alpha is set at 0.05. Therefore the null hypothesis (equality of means) cannot be denied. The two aforementioned methods showed no significant difference. On the other hand, in two procedures the comparison of variability of variances by F test was not significant for both bacteria. 

**Nonparametric analysis.** Most data did not follow the normal distribution. Therefore, nonparametric tests were preferred to exploit. Mann-Whitney is the nonparametric analog of unpaired *t*-test. It is used if the assumptions for the use of the *t-*test are not justified or called into question (e.g. in ordinal data or skewness).

**Fig. 3:**
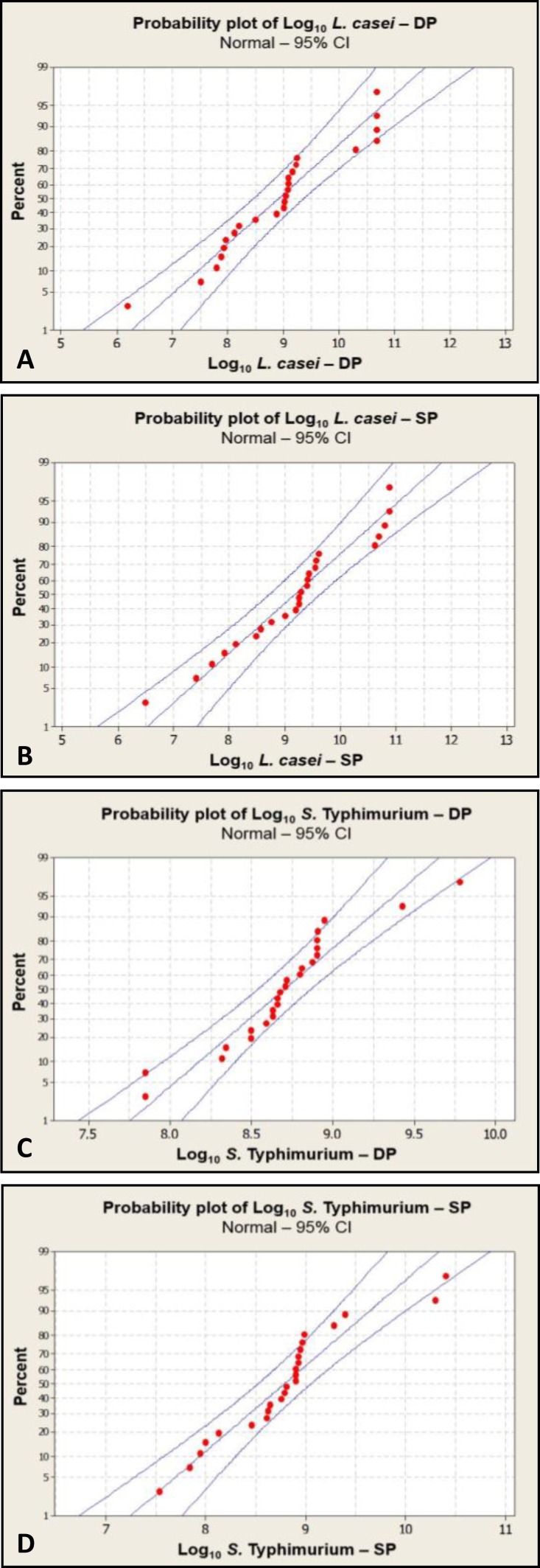
In a fit line, the data points are fitted to a line that usually does not pass through all of the data points. The middle light purple line represents a fitted distribution line that represents the trend of the data. The curved light purple lines display the approximate 95% confidence intervals for the percentiles.

Enumeration of *L. casei and S*. Typhimurium data revealed that they had approximately normal distribution. The calculated *p*-value was equivalent to 0.1363 and 0.7799, respectively. They confirmed null hypothesis in which equality of median of two methods were verified (the differences of median between two methods for both bacteria equal zero). Calculated correlation for *L. casei *and* S. *Typhimurium by two methods demonstrated that Spearman’s rho correlation coefficient (r) were 0.8673 and 0.6199 respectively at 95% significance level. H0: r = 0 it meant that there is no actual correlation, HA: r ≠ 0 it indicated that this is a correlation.^6^ The calculated *p*-values of two methods for both bacteria were dropped below 0.05, which they rejected the H0. This elucidates that the null hypothesis were rejected. Therefore, they elucidated a true relationship between two procedures for both bacteria. Figure 3 shows fitted distribution line (middle line) of DP and SP techniques for two bacteria. It is used to represent the trend and distribution pattern of data. 

**A** and **B)** related to *L. casei*, **C** and **D)** related to *S. *Typhimurium.

**Fig. 4:**
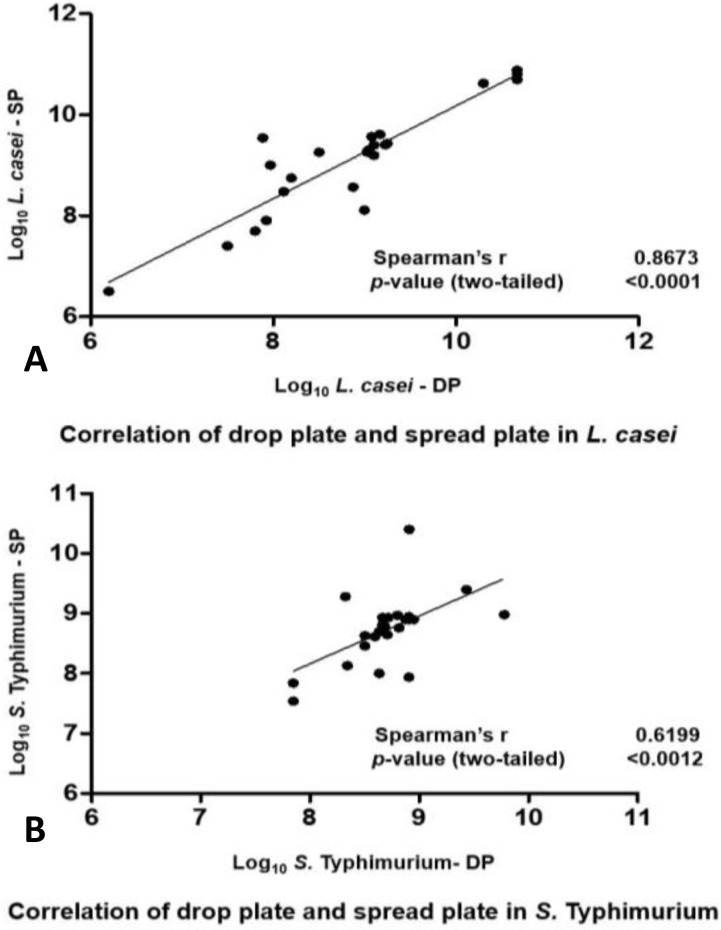
Scatter plot of DP vs. SP. **A)** *L.* *casei* and **B)** *S.* Typhimurium.

It is necessary to say that the value of the correlation coefficient alone should never consider important conclusions because the omitting outlier data influenced correlation coefficient, thus examining the respective scatterplot was always recommended. Figure 4 demonstrated the scatter plot of data in both bacteria. 

## Discussion

The most commonly used direct plating method for bacteria is the SP method. Another method, the DP method (also referred to as the spot-titer method) has been used in clinical and other settings for quantifying bacteria for many years. The results of the DP method are inter-changeable with those of the SP and double agar layer (DAL) methods by statistical analysis including analysis of variance (ANOVA) and χ2. The DP method has several advantages over the spread-plate and DAL methods: (1) it needs less time to dispense spots than to spread the microbe; (2) it uses fewer materials; (3) it requires less effort and (4) since the sample is distributed in distinct spots, colony/plaque counting is faster and less labor-intensive.^7^ The DP method can be successfully used, not only for colony-counting, but also for most probable number (MPN) enumeration.^1^ The standardized colony count method is favored over the MPN-method for routine use because of its partly higher productivity and much smaller variation in the results.^3^ The three plating methods (pour, SP, and DP techniques) are interchangeable. The DP method has been preferred because of its economy in materials and labor.^8^ Three different operators enumerated viable cells of* E. coli* using the DP and the SP methods. Statistical analyses have showed that counts obtained by the both methods were not significantly different.^9^

The improvement of science associates implying new relations between variables. The ultimate target of every research or scientific analysis is to discover relations between variables. Relations between enumeration of bacterial via DP and SP methods were surveyed by correlational research. 

For evaluation of correlation between variables, "magnitude" or "strength" as well as the significance of the correlation are vital issue. 

Correlation (r^2^) between three procedures 6×6 DP colony counts, DP, MPN and spiral plate colony counts- for *E. coli*, *Listeria monocytogenes* and *Campylobacter jejuni* were above 0.95.^1^ Spearman’s rho correlation coefficient (r) via both methods due to data distribution patterns were 0.6199 and 0.8673, respectively; which represented moderately strong and strong relation between two methods for enumeration of *S. *Typhimurium and *L. casei* respectively. Moreover, there was a significant and strong positive correlation between SP and DP procedures.

Uncertainty in repeated bacterial counting is only indirectly affected by the method in use but depends essentially on the number of counted colonies. On the other hand, the inter-laboratory uncertainty is due to fluctuation of enumeration method in use.^10^ The estimates of uncertainty are influenced by the test procedure itself, the choice of culture medium and the mathematical procedure used to derive the original ‘mean count appraisals’.^11^

Repeatability and reproducibility of every standard method have been estimated by collaborative trials and criticized published methods approve its precision for international use.^12^

For replacement of any alternative method to the corresponding reference method, it is necessary that their performance is similar to each other, without significant differences. In this respect, the ISO 16140 standard represents a key issue in producing such a method based on an inter-laboratory study.^13^

The cost of supplies, the required labor, inoculating, counting the plates, and the disposal of the relatively large volume of biohazardous waste are significant.^14^ Like any other method, this method has advantages and dis-advantages. For example, for bacteria exhibiting a swarming type of motility; e.g., *Proteus mirabilis*, *P. vulgaris*, and *Vibrio parahaemolyticus*, the DP method is not recommended because of the small size of the area covered by the drop.^3^ However, the beneficial effects include lesser time consumed, saving expenditure and faster colony counting. Above all, eight- in- one plate could have been gathered with three replications in an eight cm plate.
